# Feasibility of blood self-sampling with HemaSpot HF for Anti-*Clostridium tetani* Toxin IgG detection

**DOI:** 10.1038/s41598-025-06674-7

**Published:** 2025-07-03

**Authors:** R. Kettlitz, J. Ortmann, T. Kerrinnes, J. J. Ott, S. Castell

**Affiliations:** 1 Department for Epidemiology, Helmholtz Centre for Infection Research, Braunschweig, Lower Saxony Germany; 2 Helmholtz Centre for Infection Research (HZI), Helmholtz Institute for RNA-Based Infection Research (HIRI), Wuerzburg, Germany; 3 Medical School, Hannover, Germany; 4 PhD Programme “Epidemiology” Braunschweig-Hannover, Braunschweig, Germany; 5 German Center for Infection Research (DZIF), Braunschweig, Germany

**Keywords:** Infectious diseases, Epidemiology

## Abstract

Health research in humans often requires biosamples, e.g. blood. However, collecting venous blood samples poses multiple logistic challenges and needs to be carried by practitioners at study centres. Using self-collected samples of dried blood spots is an attractive alternative, but its feasibility remains to be determined. We investigated the feasibility (including acceptance) of a self-sampling device called HemaSpot HF (HS) employing the use case of anti-*Clostridium tetani* toxin IgG detection among 154 volunteers. Of those, n = 49 underwent an additional venous blood puncture (VBP) by medical staff (gold standard) as and control for user errors, which were determined by adding serum on HS filter paper (HemaForm (HF)). Antibody concentrations were analysed via ELISA and the serological status was determined using different cut-off values (COV). Most participants (85%) found the self-sampling using the HS device (fully) acceptable. Sensitivity for HS ranged from 70% (95% CI 0.55; 0.83; COV: 1.0 lU/mL) to 100% (95% CI 0.93; 1.00; COV: 0.01 and 0.1 lU/mL), and for HF 85% (95% CI 0.72; 0.94; 1.0 lU/mL) to 100% (95% CI 0.93; 1.00; COV: 0.01 and 0.1 lU/mL). The correlation between HS and VBP was r_Pearson_ = 0.73 (95% CI 0.56; 0.83), and between HF and VBP r_Pearson_ = 0.81 (95% CI 0.69; 0.89). The average difference in antibody concentration between HS and VBP was 1.1 IU/mL (Bland–Altman plots) and increased with the IgG concentration. Therefore, despite high acceptability, self-sampling with HS needs further improvement to be a reliable alternative to VBP.

**Supplementary Information:**

The online version contains supplementary material available at 10.1038/s41598-025-06674-7.

## Introduction

In epidemiology and medical research in general, but also especially in infectious diseases epidemiology biological samples from humans, like blood, nasal swabs or stool, are needed frequently. Especially in the case of the dynamics of population susceptibly, serological surveys assessing antibody presence or titer concentrations can help to estimate the level of immunity^1,2^. Serological data of pathogens can also be used to inform global responses in case of potential pandemic scenarios, for example with respiratory infections like SARS-CoV-2 or influenza^2,3^. However, collection, storage and completeness of biological material face logistical limitations such as time and resources rendering adequately powered studies, i.e. larger population-based studies, financially non-viable. Furthermore, the often voluntary study participation of non-symptomatic or healthy individuals is mainly based on altruistic enthusiasm that is waning^4,5^ and hence, poses challenges on recruitment and participation with an impact on study quality. One approach to solve this problem are dried blood spots (DBS). DBS have been used since 1963 to sample capillary blood from the fingertip that is applied onto a filter paper^6^. Sampling capillary blood on paper material benefits from being less invasive, requiring a smaller sampling amount, enabling shipment at ambient temperature and thereby reducing the resources and expenditures needed^7–10^, e.g. no study centre is required, and sampling can take place at any time of day that is convenient for participants. Still, most studies on DBS rely on medical staff to do the sampling. However, since further resource reduction is needed, self-sampling seems to be an inevitable solution. In a study about self-sampling capillary blood to measure the viral load of Human Immunodeficiency Virus (HIV), the acceptance and feasibility of such a device were high in the study population^11^. Approximately, 90% of participants felt comfortable collecting their blood. The experience of using the DBS kit was rated as ‘very easy’ or ‘easy’, and 98% said they would be willing to use an at-home DBS kit in the future^11^. However, DBS sampling has only been validated for a limited number of infections/pathogens such as HIV, Rubella, Measles, *Plasmodium falciparum/* *vivax* and Hepatitis B and C^12–18^. Furthermore, samples obtained through DBS have to be dried for several hours before they can be handled^9^. The blood sampling device HemaSpot HF (HS) addresses some of these limitations, by suggesting self-sampling in a standardized manner with fast drying rates^8,9^, thus,—in theory—facilitating self-sampling without study personnel and saving resources^19^. In addition, accuracy, especially sensitivity, regarding laboratory measurements compared to the gold standard of blood sampling, i.e. venous blood puncture (VBP), is important. Brooks et al. showed that staff sampled (fresh and frozen samples) DBS using HS for HIV-1 pol resistance testing in blood samples was successful in genotyping in 58% to 79% of samples depending on the cut-off values used for viral load. They detected a good concordance (86%) of resistance mutation identification of DBS to plasma^20^. Another study from 2021 investigated the diagnostic accuracy of HS compared to venous blood samples for the detection of measles- and rubella-specific immunoglobulin (Ig) G antibodies in India, showing that using the device results in a sensitivity of more than 98% for both measles and rubella IgG detection, and a specificity of 90% for measles and 98% for rubella^21^.

Up to now, only limited evidence is available on the feasibility and diagnostic performance of self-sampling with HS compared to venous blood sampling^20,22–25^. This applies for instance to the validity of anti-*Clostridium tetani* toxin IgG detection. We focused on detecting anti-*Clostridium tetani* toxin IgG antibodies due to high vaccination coverage in Germany, with 96% of children and adolescents^26^ and 54.4% of adults vaccinated within the past 10 years^27^, providing a reliable basis for antibody detection. This study aimed to investigate the feasibility of DBS self-sampling with HS, including acceptance. Furthermore, we were interested in assessing the diagnostic performance of HS in detecting of anti-*Clostridium tetani* toxin IgG as a use case for comparison with VBP as the gold standard for blood collection.

## Methods

### Study design, study population and self-sampling

This feasibility study used a convenience sample of study participants, recruited at the campus of the Helmholtz Centre for Infection Research in Brunswick, Germany. We invited 1096 individuals to participate through e-mail, flyers or during Campus meetings. Recruitment took place on selected days in January 2017; 180 individuals provided informed and written consent and were enrolled in the study. Inclusion criteria were: older than 18 years and German or English language skills. Individuals with known blood coagulation disorders (e.g. hemophilia), skin diseases on the middle or ring finger of the non-dominant hand, known immunosuppression as well as users of anticoagulant drugs were excluded. Participants received a package consisting of the HS device^8^ and sampling accessories including two lancets for fingerprick (Medipurpose SurgiLance, SLB250, 2.3 mm , 18 Gauge), study-specific instructions (S3 Appendix), study questionnaire (S4 Appendix).

The study documents were piloted before the study took place to ensure comprehensibility. For this, ten volunteers were interviewed by the study team with either the German or English study documents and asked to perform a demo sample.

A non-responder questionnaire was provided to those who denied participation (S5 Appendix).

All study participants used the device for self-sampling of capillary blood from a fingerprick. The then closed device was, together with the questionnaire and the instruction with the sampling date, returned anonymously via in-house mailing or return boxes on the campus. In addition, some study participants provided venous blood for sampling method comparison. To detect effects specific to the blood application onto the sampling device by study participants (user-error), the radial filter inserts HemaForm-80 Plate Kit (HF) used in the HS device were additionally inoculated with serum obtained through VBP by laboratory staff.

### Self-sampling device

The HS cartridge is designed for the collection and transportation of blood^8^. The box contains an absorbent filter paper with the HF innovation of a fan-shaped construction with eight sheets, covered by an application surface with a small opening for liquids. A lancet with a prick in the finger is used to provide blood. After placing 2–3 drops of blood on the application surface, the cartridge can be closed and shipped. The desiccant dries the sample^8^.

### Questionnaire on feasibility and acceptability

The questionnaire included demographic data such as sex/gender, age group, level of education and previous experience with either self-sampling or blood sampling in others. The status of education was categorized according to ISCED 2011^28^, with level 3 representing the German “Abitur”. Items assessing user-friendliness included overall self-sampling experience, assessment of the level of difficulty, evaluation of pain perception, the preferred method of blood sampling and recommendation of study participation (S4 Appendix).

### Laboratory analysis

As a use case, we determined antibody levels for anti-*Clostridium tetani* toxin IgG to assess the detectability and sensitivity of antibody levels compared to the gold standard blood sampling method. We further used a commercial assay from NovaTec^29^. Participants did not receive feedback on their laboratory results as the study was anonymized. Serum was obtained via centrifugation of whole blood in serum-specific S-Monovettes from Sarsted (Germany) according to the manufacturer’s protocol^30^. Aliquots were stored at -80 °C until further use. The radial filter inserts from the Spot-on-Science HF (F0020k, Spot-on-Science, USA) were inoculated with 80 µl of serum samples obtained as described above^8^. All extraction of dried spots was independent of the inoculation methods and was based on the manufacturer’s recommendation, with adjustments to improve reproducibility and yield. To analyze the HS samples, filter paper blades (including the middle part) are separated with sterile tweezers and scissors and transferred into a sterile Eppendorf tubes. We separated according to the following rule: two blades/tube if the filter paper was fully saturated (resulting in 4 aliquots), four blades/tube if the filter paper was half to 2/3 filled (resulting in two aliquots), and no separation if the filter paper was less than half saturated with blood (resulting in one aliquot). Subsequent extraction from the dried filter material was initialized by adding 250 μl of sterile-filtered PBS and gently shaking the tubes for 1 h at ambient temperature. The separation of re-hydrated samples in solution from the filter material was achieved by dry-spinning the prior bottom-punctured tubes inside another tube using a tabletop centrifuge at 16.000 × g for 1 min. Eluted filter extracts in the lower tube were transferred to a labelled roll-edge tube for storage at -80 °C until further usage. Antibodies against *Clostridium tetani* toxin were determined using NovaTec’s *Clostridium tetani* toxin 5S IgG plus – ELISA (PTETG043, NovaTec, Germany). All experiments were done according to the kit manufacturer’s protocol using the Tecan Nanoquant spectrophotometer, extracted filter material and serum from whole blood. The assay is calibrated against the World Health Organization International Standard for Tetanus Immunoglobulin (National Institute for Biological Standards and Control code: TE-3), using five standard concentrations: 0.0, 0.1, 0.5, 1.0, and 5.0 international units per milliliter (IU/mL). Linearity has been demonstrated across this range. Precision is acceptable, with intra-assay coefficient of variation (CV) ranging from 3.46% to 5.34% and inter-assay CV from 9.62% to 13.99%. The diagnostic sensitivity of the assay is 99.22% (95% CI 95.76–99.98%), and diagnostic specificity is 100% (95% CI 76.84–100%). Interpretation of results was based on defined cut-off values, where concentrations below 0.01 IU/mL indicate no protection, 0.01 to 0.10 IU/mL suggest no reliable immunity, 0.11 to 0.50 IU/mL indicate reliable protection with booster recommendation, 0.51 to 1.0 IU/mL suggest reliable protection with follow-up in two years, and values above 1.0 IU/mL indicate long-term protection with retesting recommended after 5 to 10 years^29^.

### Definitions and statistical analysis

Questionnaires were entered into Epi Info™ by two project team members independently and checked for consistency^31^. Discrepancies were corrected in the data set in agreement with the study management. Missing or unspecified values have been reported in the tables and labelled as such. To describe group differences in the feasibility and acceptance questions of self-sampling, we used significance tests with an alpha level of 0.05. Chi-squared test was used when frequencies were 5 or greater, and Fisher’s Exact test was used for lower frequencies. The sensitivity of the detection of tetanus was calculated from participants who provided blood samples both obtained through self-sampling and VBP.

The Net Promoter Score was calculated for assessing the degree of recommendation of study participation according to Reichheld et al.^32^. Participants were asked whether they would recommend acquaintances to participate in a study with self-sampling of blood. The score was calculated from the percentage of those who recommend participation (promoters) minus those who do not recommend participation in such a study (detractors). A higher value indicates a stronger recommendation. A Net Promoter Score of > 0 is good, a score of > 50 is excellent and a score of negative values is interpreted as “not recommended”^33^.

Further, we calculated sensitivity of HS compared to VBP and HF compared to VBP. Pearson correlation coefficient was calculated for the comparison of HS versus VBP and HF versus VBP based on anti-*Clostridium tetani* toxin IgG concentration (IU/mL). In addition, Bland–Altman plots, as a graphical representation of the relationships between the different sampling strategies, were used. Introduced by Altman and Bland in 1983, Bland–Altman plots provide a method for assessing the agreement between two quantitative measurement methods that evaluate the same parameter. The plot displays the difference between paired measurements on the y-axis versus their average on the x-axis. The plot includes the mean difference (bias) and the limits of agreement, which are defined as the mean difference plus or minus 1.96 times the standard deviation of the differences. This visualization is useful for detecting systematic bias; ideal results show differences close to zero along the y-axis^34,35^. As sensitivity analysis, we also compared antibody concentration in HS compared to HF. We used R [version 4.3.2.] for all statistical analyses^36^. The R script can be retrieved from elsewhere (DOI: 10.5281/zenodo.14592165). We used OpenAI’s ChatGPT v2 to review and refine the R code for improved structure and functionality^37^. For clarity and precision, the English language parts of this manuscript have been edited using DeepL Pro and DeepL Write^38^. The authors checked the output for correctness and took responsibility for it.

### Ethics and data protection

The study was reviewed and approved by the ethics committee of Hannover Medical School by June 21, 2016 (No: 3251–2016). A data protection concept was developed and approved by the internal data protection officer. The Federal Commissioner for Data Protection and Freedom of Information advised on the study. The study was performed in alignment with the Declaration of Helsinki [except study registration]. In addition, the study follows the recommendations for ensuring good epidemiological practice of the German Society for Epidemiology^39^.

## Results

### Participation

**Fig. 1:**
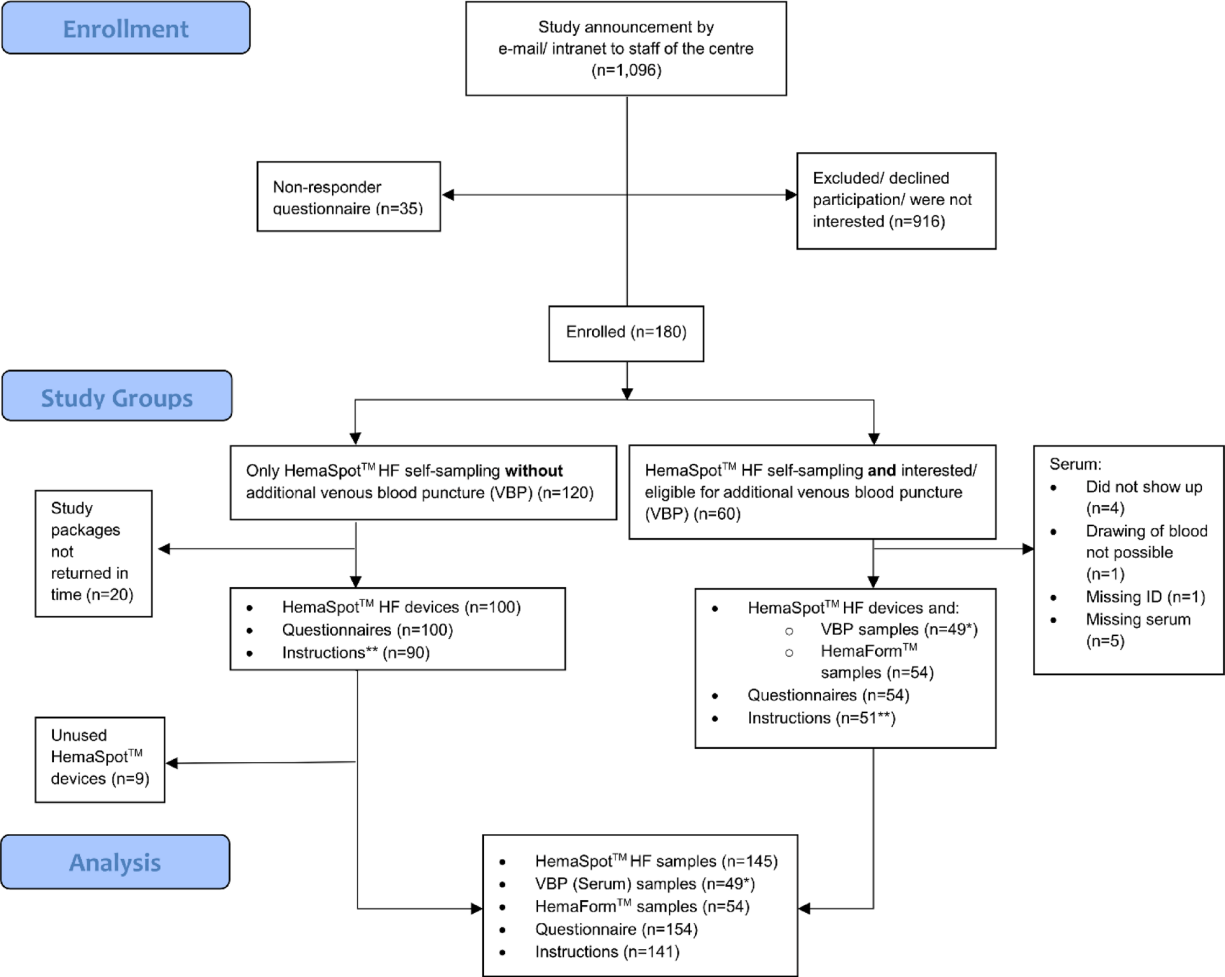
Flow chart of study participant selection and participation. *The n can vary for each test depending on how much blood was recovered from one samples; **instructions contain sampling date.

We enrolled 180 voluntary participants in the study of which 154 returned the study packages in time (Fig. 1). A sub-sample of 49 participants were eligible for an analysis for comparison of gold standard with self-sampling. The study population consisted of 58% females, 31% under the age of 30, and 18% were over the age of 49. An International Standard Classification of Education (ISCED) level 3, equals German “Abitur”, was reported from 84%. Overall, 49% of the participants stated having experience with blood-sampling (Table 1). Additionally, 35 individuals filled out the non-responder form: The most stated reasons for nonparticipation were inhibitions regarding drawing blood samples (37%; 13/35), being afraid of blood self-sampling (37%; 13/35) and health restrictions that prevent participation (26%; 9/35) (S2 Appendix). After arrival in the laboratory, 77% (119/154) of the received devices showed no conspicuous features. Overall, 20/35 (57%) showed internal conspicuous features. Such features were mostly related to light blood spatter on the rubber (25% of all internal particularities). There were no internal or external damages on the devices registered.

**Table 1:** Characteristics of study population.

Characteristics study population	N = 154^a,b^	N = 49^a,c^
Age category		
< 30	48 (31%)	13 (27%)
30–49	78 (51%)	28 (57%)
≥ 50	28 (18%)	8 (16%)
Missing	0	0
Gender/sex		
Female	90 (58%)	30 (61%)
Male	64 (42%)	19 (39%)
Missing	0	0
Education		
Lower than ISCED level 3	21 (14%)	7 (15%)
ISCED level 3	129 (84%)	40 (83%)
Don’t know	3 (2%)	1 (2%)
Missing	1	1
Experience with blood sampling		
No	78 (51%)	22 (45%)
Yes	76 (49%)	27 (55%)
Missing	0	0

^a^n (%);*classified according to ISCED (international standard classification of education); level 3 equals German “Abitur^”;^

^b^Study population which performed self-sampling and filled out the questionnaires on feasibility and acceptance.

^c^Study population with additional venous blood puncture.

### Feasibility including acceptance of blood sampling

**Fig. 2:**
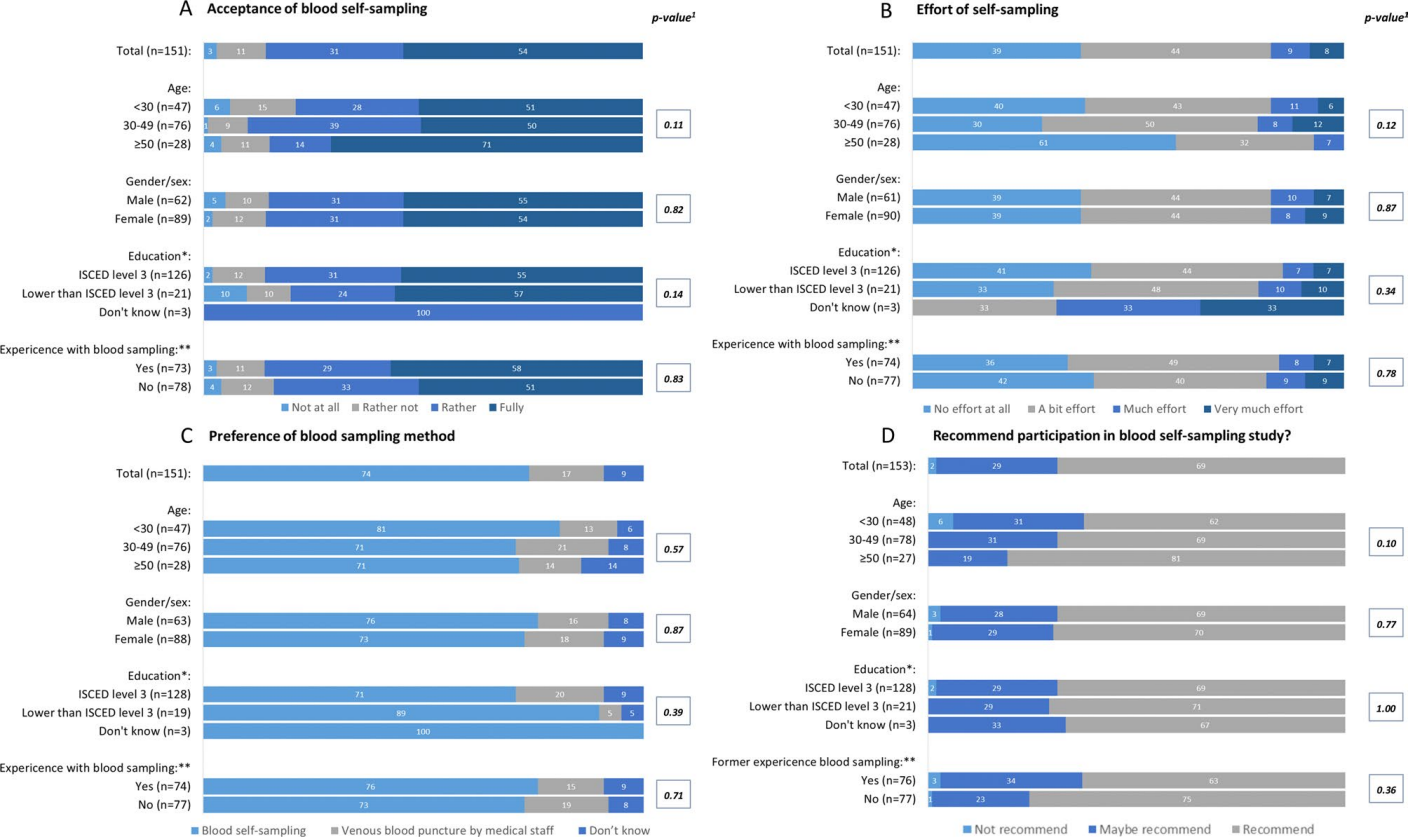
Study participants’ characteristics (shown in percentage) of self-blood sampling acceptance within the question: (**A**) “To what extent do you agree that collecting the blood sample yourself was acceptable?”. (**B**) “How much effort did it take for you to take blood from yourself?”. (**C**) “Assuming that the laboratory result of self-sampling is the same as that of venous blood sampling by medical staff, which method would you prefer and why?”. (**D**) “Would you recommend that your friends take part in a study and collect their own blood samples?”—stratified by age, gender, education, and experience with blood sampling; *classified according to ISCED (International Standard Classification of Education); level 3 equals German “Abitur”- one missing value in this category; **former experience with blood sampling (Have you ever had blood taken before this study?); ^1^significance tests for group differences were conducted with an alpha level of 0.05. Chi-squared test was used when frequencies were 5 or greater, and for lower frequencies, Fisher’s Exact test was used.

A total of 128/151 (85%) found the self-sampling using HS rather to fully acceptable (Fig. 2A). In those who rated the self-sampling as fully acceptable 59% (48/82) were female. The highest acceptance proportion was found in those 30–49 years old (46%; 38/82). A proportion of 44% (67/141) reported having to exert themselves somewhat during self-sampling. Within those older than 49 years, 61% (17/28) reported not having to apply any effort while self-sampling (Fig. 2B). Self- sampling instead of VBP at a study centre was preferred by 74% (112/151) of the respondents (Fig. 2D). Of those 17% (26/151), who preferred a VBP by medical staff over blood self-sampling, 62% (16/26) were female , between 30 to 49 years old (62%; 16/26), and had no experience with blood sampling (58%; 15/26). In the group that preferred self-sampling, the most common reason given was that it saves time in travelling, appointments and waiting (66%; 63/95). In those who preferred VBP by medical staff, the most common reason was that VBP was easy and uncomplicated (39%; 9/23) or that it gave a sense of security when done by trained staff (22%; 5/23). In 69% (106/153) of cases, respondents would recommend participating in a study for blood self-sampling to their friends (Fig. 2C). The Net Promoter Score was excellent at 95%. The highest levels of recommendation were found among women and those with no previous experience of blood sampling respectively, at 97%, and among the two oldest age groups and those with education below ISCED (International Standard Classification of Education) level 3 respectively, at 100%. Almost every one of the participants answered the question on whether the pain was reasonable for them in the context of this study with partially reasonable (98%; 150/153). On a scale from 0 (no pain at all) to 10 (worst pain imaginable), the respondents rated a median of 1 (IQR: 1–3) for the pain caused by self-sampling. At least one approach to increase blood flow before self-sampling was used by 90% (138/154) of the participants. “Massaging” being the most frequently used method in this context (66%; 102/154). Wiping away the first drop of blood as requested in the instructions was reported by 94% (144/154). The targeted amount of blood drops, i.e. 3, needed to fill the filter paper was achieved by 33% (48/145). Whereas 46% (67/145) of the participants provided more than 3 drops. Two lancets for self-sampling capillary blood were used by 55% (84/153). Consequences like prolonged post-puncture bleeding were reported by 12% (18/150) of participants. Of all responders, 92% (142/150) found the manual to be easily understandable. No significant differences between the groups were found. Detailed information can be retrieved from the S1 Appendix.

### Feasibility of Anti-*Clostridium tetani* Toxin IgG detection

When lower cut-off values of 0.01 and 0.1 lU/mL were applied when dividing the anti-*Clostridium tetani toxin* IgG concentration in negative and positive, sensitivity was calculated as 100% (0.93, 1.00) for both HS and HF. Stricter cut-off values resulted in lower sensitivity for HF (85%; 95 CI 0.72; 0.94) as well as for HS (70%; 95 CI 0.55; 0.83) (Table 2).

**Table 2:** Validity and reliability of laboratory results for analyses of anti-*Clostridium tetani* toxin IgG.

Cut-off value (lU/mL)*	Tests (n = 49)	Serum: test +	Serum: test −	Total	Sensitivity % (95%-CI)
0.01	HemaSpot HF vs. serum (VBP)				
	HemaSpot: test +	49	0	49	100% (0.93, 1.00)
	HemaSpot: test −	0	0	0	
	Total	49	0	49	
	HemaForm vs. serum (VBP)				
	HemaForm: test +	49	0	49	100% (0.93, 1.00)
	HemaForm: test −	0	0	0	
	Total	49	0	49	
0.1	HemaSpot HF vs. serum (VBP)				
	HemaSpot: test +	49	0	49	100% (0.93, 1.00)
	HemaSpot: test −	0	0	0	
	Total	49	0	49	
	HemaForm vs. serum (VBP)				
	HemaForm: test +	49	0	49	100% (0.93, 1.00)
	HemaForm: test −	0	0	0	
	Total	49	0	49	
0.5	HemaSpot HF vs. serum (VBP)				
	HemaSpot: test +	46	0	46	94% (0.83, 0.99)
	HemaSpot: test −	3	0	3	
	Total	49	0	49	
	HemaForm vs. serum (VBP)				
	HemaForm: test +	49	0	49	100% (0.93, 1.00)
	HemaForm: test −	0	0	0	
	Total	49	0	49	
1.0	HemaSpot HF vs. serum (VBP)				
	HemaSpot: test +	33	0	33	70% (0.55, 0.83)
	HemaSpot: test −	14	2	16	
	Total	47	2	49	
	HemaForm vs. serum (VBP)				
	HemaForm: test +	40	0	40	85% (0.72, 0.94)
	HemaForm: test −	7	2	9	
	Total	47	2	49	

*Cut-off values and interpretations according to the official manual of the manufacturer: 0.01 lU/mL = no protective antibody level, 0.1 lU/mL = no reliable protection (booster and control after 4–6 weeks recommended), 0.5 lU/mL = reliable protection (booster and control after 4–6 weeks recommended), 1.0 lU/mL = reliable protection (control after 2 years recommended, booster not required) [NovaTec’s *Clostridium tetani* toxin 5S IgG plus – ELISA (PTETG043, NovaTec, Germany)], VBP = venous blood puncture.

**Fig. 3:**
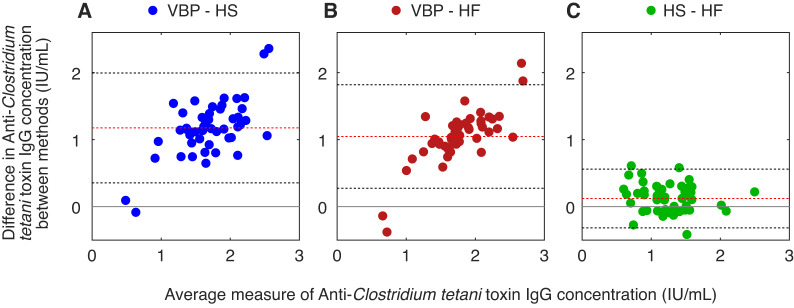
Bland–Altman plot for anti-*Clostridium tetani* toxin IgG concentration (IU/mL). (**A**) Venous blood puncture (VBP) by medical professionals) vs. HemaSpot HF (HS) (self-sampling): differences were calculated by subtracting measured anti-*Clostridium tetani* toxin IgG concentrations found in HS from those found in serum. (**B**) Venous blood puncture (VBP) by medical professionals vs. HemaForm (HF): differences were calculated by subtracting measured anti-*Clostridium tetani* toxin IgG concentrations found in HF from those found in serum. (**C**) HemaSpot HF (HS) (self-sampling) vs. HemaForm (HF): differences were calculated by distracting measured anti-*Clostridium tetani* toxin IgG concentrations found in HF from those found in HS. Y-axis: The difference of two measurements for every individual is depicted. The grey line at zero demonstrates the theoretical perfect agreement, i.e. both methods would yield the same value for each measurement regardless of method, the red dashed line shows the actual mean of the difference for the two methods that are compared demonstrating the average bias, and the black dotted lines are the 95% limits of agreement with the actual mean as centre. X-axis: the mean of both measurement for every individual is depicted.

The correlation between the different antibody concentration measurement (IU/mL) was assessed to be moderate to strong: Comparing HS to VBP we estimated r_Pearson_ = 0.73 (95% CI 0.56; 0.83) and comparing HF to VBP r_Pearson_ = 0.81 (95% CI 0.69; 0.89). In the Bland–Altman plots (Fig. 3) the comparison of self-sampling to VBP can be seen based on deviations of the mean difference over mean concentration levels. The mean difference of measured IgG concentration exceeds 1.0 when comparing HS or HF to VBP (Fig. 3 A,B). This indicates that the mean difference deviates from the expected value, i.e. zero. On average the IgG concentration for tetanus is higher in serum collected with VBP. It is noticeable that the two downward outliers, i.e. where the concentrations appear to be higher in self-sampling, occur in the lower measuring range (mean of the two measurements), while the outliers with higher concentrations in sera from VBP occur in the higher average measuring range. Comparing the IgG concentration between HS and HF, we observe that the average mean difference is closer to zero (Fig. 3C).

## Discussion

We conducted a field-based epidemiologic study among voluntary participants to pilot a device which claims to be a suitable and practical option for blood self-sampling. We found a high acceptance, high recommendation and less pain of this self-sampling device. Participants showed a strong preference for self-sampling over visiting the study centre for blood sampling, i.e. 74% (112/151) preferred blood self-sampling versus 17% (26/151) VBP by medical staff. The most frequently given reason was the time saving aspect. The Net Promoter Score reached an excellent value of 95% in the study population. A value closer to 100% indicates a stronger recommendation^32^. Pain during the self-sampling process was negligible as it was being mainly rated at 1 on a scale from 0 (no pain at all) to 10 (worst pain imaginable). Further, most participants would prefer self-sampling instead of VBP at a study centre. High acceptance of self-sampling with DBS cards can be found similarly in the literature. A study, conducted in the United States, found in a population with 153 participants that over 84% reported that the collection, packaging and shipping of self-collection of saliva or blood were acceptable^40^. Acceptance of HS was also confirmed by diabetic patients (n = 128) previously with a 61% preference of using HS compared to going to their general practitioner^22^. Another study conducted with HemaSpot SD, an advancement of HS designed to collect a larger blood volume, found similar high values of feasibility and acceptance^11^. Approximately 90% reported that they felt comfortable with self-sampling their blood, and using the HemaSpot SD was rated as ‘very easy’ or ‘easy’, and 98% said they would be willing to use such an at-home self-sampling kit in the future^11^. Despite high overall acceptance and recommendation, we have shown that there was still some effort for the participants to perform the self-sampling involved. Most non-responders stated that they have inhibitions or are even afraid of drawing blood on their own. Regarding reasons for not participating, none of the people named data protection concerns or a lack of trust as a reason. In our study sample over 50% of the respondents needed more than one lancet for fingerprick to draw capillary blood, which might also influence the processing and feasibility of the self-sampling. Using more than one lancet to draw blood might increase the pain for the participant during the sampling process, but it also might increase the risk of wound infection due to two fingerpricks instead of one. Overall, 138/151 (90%) participants used at least one method to improve blood circulation before taking the blood sample; 102/151 (66%) participants used the technique of finger massage. Therefore, we recommend to test a larger lancet in the pilot phase of self-sampling projects than we used to achieve a deeper prick in the finger (Medipurpose SurgiLance, SLB250, 2.3 mm, 18 Gauge). In the group of people who preferred VBP, 62% were female (16/26), 62% were aged 30–49 years (16/26), and 58% had no experience of blood sampling (15/26). Individuals with these characteristics need specific support in self-sampling to avoid selection bias in such studies, for example support videos that specifically target having no experience with drawing blood.

The second component of our study investigated the diagnostic performance in detecting antibodies against infectious agents based on the use case of anti-*Clostridium tetani* toxin IgG. For these antibodies, the Pearson correlation coefficient in the samples for HS compared with serum from VBP was moderate and for HF compared with VBP slightly stronger, which could be explained by the sampling process of self-sampling capillary blood by participants compared with serum used for HF and dripped on the filter paper in the laboratory by medical staff, which is more comparable to serum trough VBP. The sensitivities of HS ranged, depending on the cut-off values used, between 70 and 100%, and slightly higher for HF. The lower the cut-off values were, the higher the sensitivities were for HS and HF. The higher sensitivities in the lower measuring range might be explained due to the high IgG concentration itself. When interpreting the different cut-off values, it is important to consider that DBS tend to show lower antibody levels than serum in our study. Sensitivity in HS (vs. VBP) is 100% at lower cut-off values and gradually decreases starting from 0.5 IU/mL. In sero-epidemiological studies usually this cut-off is used, when aiming to demonstrate long-term or strong protective immunity^41,42^. Hence, the limitation in sensitivity with high cut-off values plays no practical role in population-based research. Thus, self-sampling had lower sensitivity values for measuring antibody levels deemed necessary for long-term protection. Self-sampling devices must ensure enough blood volume in a useful concentration is provided for laboratory analyses. Since sensitivities of both HS and HF were slightly different with HS faring worse, we cannot exclude a small impact from user error or logistical problems during storage or shipment of the device. Similar to our results, another study using DBS cards (whole blood) and compared it to serum from VBP, both sampled by medical professionals, also showed high sensitivity of detection of anti-*Clostridium tetani* toxin IgG [100% (95% CI 0.99, 1.00)]^43^. Other studies conducted with HS also found high sensitivities of around 98% for rubella and measles when comparing HS sampling of capillary blood to serum collected with VBP (for HS and serum through medical staff)^21^. The visualization of the difference in the measurements with Bland–Altman plots showed a systematic measurement error comparing both HS and HF with VBP (Fig. 3 A,B). With higher antibody concentration the difference between the measures increased for both comparison as well. This error was not seen, when comparing the measurements of HS with HF directly (Fig. 3C); allowing the interpretation that the filterpaper in the device might cause the deviation and not the self-sampling as such. The differences from both HS and HF to the gold standard could also be explained by the processing of the DBS in the laboratory itself. Plots showed a different picture than sensitivity values, especially at lower cut-offs. IgG measurements were categorized as seropositive and seronegative to estimate sensitivity. High antibody concentration per lU/mL in the study population could lead to a false expectation. To reduce ELISA misclassification, we recommend that future studies consider normalisation, e.g. by haemoglobin, to reduce serum-specific background noise^44^. Given the differential profile of infectious agents, it is also crucial to specify the pathogens for which such an application has been validated and that they may not be universally applicable, especially not for viruses with fast weaning antibody responses, since the sensitivity as defined by the amount of available antibodies in the biospecimen. In addition, conclusions from the Bland–Altman plots suggested an increasing bias between VBP/HS and VBP/HF at higher IgG concentrations. Additional data, especially at the upper concentration ranges, are needed to confirm this trend and better understand the underlying causes.

The main strength of our study is the prevalidation of study documents and the investigation of a vaccine-preventable disease, as well as the analysis of the reasons for non-participation in serological studies with a self-sampling device. One drawback of the study results from the potentially selective nature of study participants. This study had several limitations. Firstly, the sample size was relatively small and confined to a single geographic area in Germany, which may restrict the applicability of the findings to other population groups Additionally, the number of participants above the age of 50 was limited, which compromises the reliability of age-specific inferences. The sample population also lacks representativeness, as participants were recruited from a research center specializing in infectious diseases, resulting in approximately half of the participants having prior experience with blood draws. Regarding the laboratory analyses, our approach offers only one use case to demonstrate basic feasibility and to identify challenges; other laboratory analyses might show specific problems of their own.

While our findings demonstrated that DBS sampling is well accepted and offers many advantages, it is important to note that there are also limitations. In particular, laboratory analysis of DBS can be more complex and time-consuming than the analysis of traditional venous blood samples, due to limited laboratory experience and the absence of standardized workflows^45^. Meanwhile Spot-On-Sciences developed another sampling device called HemaSpot HD, which allow for the collection of higher volume, up to 160 μL, and more punches possible for further analysis compared to HemaSpot HF and HemaSpot SE, which allows the separation of whole blood into its components, the whole blood cells and the serum^46^. Nevertheless, our results still have implications and call for new approaches and technological inventions to make DBS blood sampling not only an alternative to VBP. As long as DBS approaches still require assisting medical staff they are no valid alternative for VBP in study centres. Cost differences between DBS self-sampling with HS and traditional VBP were not assessed in this feasibility study. Further research is needed to evaluate the cost-effectiveness of DBS-based approaches, especially when used in large sero-epidemiological studies.

Due to the high acceptance of self-sampling with such a device, we see a future application in sero-epidemiological studies for blood collection. However, the sampling device needs further improvement to be a reliable alternative to the gold standard VBP.

## Electronic supplementary material

Below is the link to the electronic supplementary material.

Supplementary Material 1.

## Data Availability

Aggregated data is presented in the figures and tables of the manuscript. Due to data protection concerns, access to the individual-level data is restricted. If you have any questions or require further information, please contact the authors at Stefanie.Castell@helmholtz-hzi.de.
