# Supplementary material for: Feasibility of blood self-sampling with HemaSpot HF for Anti-Clostridium tetani Toxin IgG detection
Source: Sci Rep. 2025 Jul 3;15:23693. doi: 10.1038/s41598-025-06674-7 (PMC12223160; doi:10.1038/s41598-025-06674-7)
Supplement: Supplementary file 1 — Supplementary Material 1. [file 41598_2025_6674_MOESM1_ESM.docx]

Supplement Material

S1 Appendix Table 1. Study population characteristics

| **Characteristic** | **Overall** | **Stratification** | | | | | | | | | |
| --- | --- | --- | --- | --- | --- | --- | --- | --- | --- | --- | --- |
|  |  | **Age category**  **(N=154)** | | | **Gender**  **(N=154)** | | **Education**  **(N=153)** | | | **Experience with blood sampling^2^**  **(N=154)** | |
|  | N = 154*^1^*  (100%) | **<30** N = 48*^1^* | **30-49** N = 78*^1^* | **≥50** N = 28*^1^* | **Female** N = 90^1^ | **Male** N = 64^1^ | **Lower than ISCED level 3** N = 21^1^ | **ISCED level 3** N = 129^1^ | **Don't know** N = 3^1^ | **No**  N = 78*^1^* | **Yes** N = 76*^1^* |
| **To what extent do you agree with the following statements? The procedure of blood self-sampling was acceptable.** | | | | | | | | | | | |
| Fully | 82 (100%) | 24 (29%) | 38 (46%) | 20 (24%) | 48 (59%) | 34 (41%) | 12 (15%) | 69 (85%) | 0 (0%) | 40 (49%) | 42 (51%) |
| Rather | 47 (100%) | 13 (28%) | 30 (64%) | 4 (8.5%) | 28 (60%) | 19 (40%) | 5 (11%) | 39 (83%) | 3 (6.4%) | 26 (55%) | 21 (45%) |
| Rather not | 17 (100%) | 7 (41%) | 7 (41%) | 3 (18%) | 11 (65%) | 6 (35%) | 2 (12%) | 15 (88%) | 0 (0%) | 9 (53%) | 8 (47%) |
| Not at all | 5 (100%) | 3 (60%) | 1 (20%) | 1 (20%) | 2 (40%) | 3 (60%) | 2 (40%) | 3 (60%) | 0 (0%) | 3 (60%) | 2 (40%) |
| Unknown | 3 | 1 | 2 | 0 | 1 | 2 | 0 | 3 | 0 | 0 | 3 |
| **To what extent do you agree with the following statements? The procedure of blood self-sampling was user-friendly.** | | | | | | | | | | | |
| Fully | 54 (100%) | 12 (22%) | 30 (56%) | 12 (22%) | 33 (61%) | 21 (39%) | 8 (15%) | 45 (85%) | 0 (0%) | 25 (46%) | 29 (54%) |
| Rather | 61 (100%) | 24 (39%) | 29 (48%) | 8 (13%) | 35 (57%) | 26 (43%) | 9 (15%) | 49 (80%) | 3 (4.9%) | 33 (54%) | 28 (46%) |
| Rather not | 29 (100%) | 8 (28%) | 15 (52%) | 6 (21%) | 17 (59%) | 12 (41%) | 3 (10%) | 26 (90%) | 0 (0%) | 12 (41%) | 17 (59%) |
| Not at all | 8 (100%) | 3 (38%) | 3 (38%) | 2 (25%) | 5 (63%) | 3 (38%) | 1 (13%) | 7 (88%) | 0 (0%) | 8 (100%) | 0 (0%) |
| Unknown | 2 | 1 | 1 | 0 | 0 | 2 | 0 | 2 | 0 | 0 | 2 |
| **How would you rate your overall experience with blood self-sampling on a scale from 0 (very negative) to 10 (very positive)?** | | | | | | | | | | | |
|  | 7.00 (5.00, 8.00) | 6.00 (5.00, 8.00) | 7.00 (5.00, 8.00) | 8.00 (5.00, 8.50) | 7.50 (5.00, 9.00) | 6.50 (5.00, 8.00) | 8.00 (7.00, 9.00) | 7.00 (5.00, 8.00) | 6.00 (5.00, 8.00) | 6.50 (5.00, 8.00) | 8.00 (5.50, 8.00) |
| Unknown | 0 | 0 | 0 | 0 | 0 | 0 | 0 | 0 | 0 | 0 | 0 |
| **How much effort did it imply for you, to draw blood self-sampling?** | | | | | | | | | | | |
| Very much effort | 12 (100%) | 3 (25%) | 9 (75%) | 0 (0%) | 8 (67%) | 4 (33%) | 2 (17%) | 9 (75%) | 1 (8.3%) | 7 (58%) | 5 (42%) |
| Much effort | 13 (100%) | 5 (38%) | 6 (46%) | 2 (15%) | 7 (54%) | 6 (46%) | 2 (17%) | 9 (75%) | 1 (8.3%) | 7 (54%) | 6 (46%) |
| A bit effort | 67 (100%) | 20 (30%) | 38 (57%) | 9 (13%) | 40 (60%) | 27 (40%) | 10 (15%) | 56 (84%) | 1 (1.5%) | 31 (46%) | 36 (54%) |
| No effort at all | 59 (100%) | 19 (32%) | 23 (39%) | 17 (29%) | 35 (59%) | 24 (41%) | 7 (12%) | 52 (88%) | 0 (0%) | 32 (54%) | 27 (46%) |
| Unknown | 3 | 1 | 2 | 0 | 0 | 3 | 0 | 3 | 0 | 1 | 2 |
| **Use of the prick device was …** | | | | | | | | | | | |
| Very difficult | 5 (100%) | 2 (40%) | 2 (40%) | 1 (20%) | 2 (40%) | 3 (60%) | 1 (20%) | 4 (80%) | 0 (0%) | 3 (60%) | 2 (40%) |
| Difficult | 6 (100%) | 2 (33%) | 4 (67%) | 0 (0%) | 4 (67%) | 2 (33%) | 1 (20%) | 2 (40%) | 2 (40%) | 3 (50%) | 3 (50%) |
| Easy | 55 (100%) | 23 (42%) | 23 (42%) | 9 (16%) | 30 (55%) | 25 (45%) | 4 (7.3%) | 51 (93%) | 0 (0%) | 27 (49%) | 28 (51%) |
| Very easy | 86 (100%) | 21 (24%) | 47 (55%) | 18 (21%) | 53 (62%) | 33 (38%) | 15 (17%) | 70 (81%) | 1 (1.2%) | 45 (52%) | 41 (48%) |
| Unknown | 2 | 0 | 2 | 0 | 1 | 1 | 0 | 2 | 0 | 0 | 2 |
| **Overall, the blood self-sampling was ….** | | | | | | | | | | | |
| Very difficult | 9 (100%) | 2 (22%) | 4 (44%) | 3 (33%) | 5 (56%) | 4 (44%) | 0 (0%) | 9 (100%) | 0 (0%) | 7 (78%) | 2 (22%) |
| Difficult | 28 (100%) | 10 (36%) | 14 (50%) | 4 (14%) | 19 (68%) | 9 (32%) | 2 (7.1%) | 25 (89%) | 1 (3.6%) | 15 (54%) | 13 (46%) |
| Easy | 84 (100%) | 28 (33%) | 43 (51%) | 13 (15%) | 45 (54%) | 39 (46%) | 11 (13%) | 71 (86%) | 1 (1.2%) | 45 (54%) | 39 (46%) |
| Very easy | 32 (100%) | 8 (25%) | 16 (50%) | 8 (25%) | 21 (66%) | 11 (34%) | 8 (25%) | 24 (75%) | 0 (0%) | 11 (34%) | 21 (66%) |
| Unknown | 1 | 0 | 1 | 0 | 0 | 1 | 0 | 0 | 1 | 0 | 1 |
| **Please rate the level of difficulty of the blood self-sampling. Applying the blood to the filter was** | | | | | | | | | | | |
| Very difficult | 40 (100%) | 17 (43%) | 16 (40%) | 7 (18%) | 22 (55%) | 18 (45%) | 2 (5.0%) | 37 (93%) | 1 (2.5%) | 24 (60%) | 16 (40%) |
| Difficult | 54 (100%) | 21 (39%) | 28 (52%) | 5 (9.3%) | 33 (61%) | 21 (39%) | 7 (13%) | 46 (87%) | 0 (0%) | 24 (44%) | 30 (56%) |
| Easy | 45 (100%) | 8 (18%) | 28 (62%) | 9 (20%) | 25 (56%) | 20 (44%) | 8 (18%) | 35 (78%) | 2 (4.4%) | 23 (51%) | 22 (49%) |
| Very easy | 10 (100%) | 0 (0%) | 3 (30%) | 7 (70%) | 7 (70%) | 3 (30%) | 4 (40%) | 6 (60%) | 0 (0%) | 5 (50%) | 5 (50%) |
| Unknown | 5 | 2 | 3 | 0 | 3 | 2 | 0 | 5 | 0 | 2 | 3 |
| **Assuming the laboratory results of the blood self-sampling would be equal to those of the venous blood puncture by medical staff, which method of blood collection would you prefer and why?** | | | | | | | | | | | |
| Blood self-sampling | 112 (100%) | 38 (34%) | 54 (48%) | 20 (18%) | 64 (57%) | 48 (43%) | 17 (15%) | 91 (82%) | 3 (2.7%) | 56 (50%) | 56 (50%) |
| Don't know | 13 (100%) | 3 (23%) | 6 (46%) | 4 (31%) | 8 (62%) | 5 (38%) | 1 (7.7%) | 12 (92%) | 0 (0%) | 6 (46%) | 7 (54%) |
| Venous blood puncture by medical staff | 26 (100%) | 6 (23%) | 16 (62%) | 4 (15%) | 16 (62%) | 10 (38%) | 1 (3.8%) | 25 (96%) | 0 (0%) | 15 (58%) | 11 (42%) |
| Unknown | 3 | 1 | 2 | 0 | 2 | 1 | 2 | 1 | 0 | 1 | 2 |
| **Prefer blood self-sampling, because …..** | | | | | | | | | | | |
| I can determine the time and place myself | 16 (100%) | 9 (56%) | 6 (38%) | 1 (6.3%) | 7 (44%) | 9 (56%) | 1 (6.3%) | 14 (88%) | 1 (6.3%) | 10 (63%) | 6 (38%) |
| I don't like going to the doctor | 1 (100%) | 1 (100%) | 0 (0%) | 0 (0%) | 1 (100%) | 0 (0%) | 0 (0%) | 1 (100%) | 0 (0%) | 1 (100%) | 0 (0%) |
| I like to take responsibility for my own care | 1 (100%) | 1 (100%) | 0 (0%) | 0 (0%) | 0 (0%) | 1 (100%) | 0 (0%) | 1 (100%) | 0 (0%) | 0 (0%) | 1 (100%) |
| I make the blood sample less painful | 3 (100%) | 0 (0%) | 3 (100%) | 0 (0%) | 3 (100%) | 0 (0%) | 1 (33%) | 2 (67%) | 0 (0%) | 0 (0%) | 3 (100%) |
| It is simple and straightforward | 5 (100%) | 2 (40%) | 3 (60%) | 0 (0%) | 4 (80%) | 1 (20%) | 0 (0%) | 5 (100%) | 0 (0%) | 2 (40%) | 3 (60%) |
| It makes me feel safe to do things myself | 2 (100%) | 1 (50%) | 1 (50%) | 0 (0%) | 0 (0%) | 2 (100%) | 1 (50%) | 1 (50%) | 0 (0%) | 1 (50%) | 1 (50%) |
| less time is needed (travel, appointments/waiting time) | 63 (100%) | 16 (25%) | 31 (49%) | 16 (25%) | 36 (57%) | 27 (43%) | 12 (19%) | 50 (79%) | 1 (1.6%) | 33 (52%) | 30 (48%) |
| The risk of infection is lower | 4 (100%) | 2 (50%) | 2 (50%) | 0 (0%) | 4 (100%) | 0 (0%) | 0 (0%) | 3 (75%) | 1 (25%) | 1 (25%) | 3 (75%) |
| Unknown | 59 | 16 | 32 | 11 | 35 | 24 | 6 | 52 | 0 | 30 | 29 |
| **Prefer venous blood puncture by medical staff, because** | | | | | | | | | | | |
| I am afraid of doing something wrong | 2 (100%) | 2 (100%) | 0 (0%) | 0 (0%) | 2 (100%) | 0 (0%) | 0 (0%) | 2 (100%) | 0 (0%) | 1 (50%) | 1 (50%) |
| I do not want to hurt myself | 4 (100%) | 0 (0%) | 4 (100%) | 0 (0%) | 2 (50%) | 2 (50%) | 0 (0%) | 4 (100%) | 0 (0%) | 1 (25%) | 3 (75%) |
| It gives me a sense of security when it is done by trained personnel | 5 (100%) | 1 (20%) | 3 (60%) | 1 (20%) | 4 (80%) | 1 (20%) | 0 (0%) | 5 (100%) | 0 (0%) | 4 (80%) | 1 (20%) |
| It is simple and uncomplicated | 9 (100%) | 2 (22%) | 5 (56%) | 2 (22%) | 3 (33%) | 6 (67%) | 1 (11%) | 8 (89%) | 0 (0%) | 6 (67%) | 3 (33%) |
| It takes less time (blood sample) | 1 (100%) | 0 (0%) | 1 (100%) | 0 (0%) | 0 (0%) | 1 (100%) | 0 (0%) | 1 (100%) | 0 (0%) | 0 (0%) | 1 (100%) |
| When it is done by trained personnel, the blood sample causes less pain | 2 (100%) | 1 (50%) | 1 (50%) | 0 (0%) | 2 (100%) | 0 (0%) | 0 (0%) | 2 (100%) | 0 (0%) | 1 (50%) | 1 (50%) |
| Unknown | 131 | 42 | 64 | 25 | 77 | 54 | 20 | 107 | 3 | 65 | 66 |
| **Would you recommend participation in a study for blood self-sampling to your friends?** | | | | | | | | | | | |
| Maybe recommend | 44 (100%) | 15 (34%) | 24 (55%) | 5 (11%) | 26 (59%) | 18 (41%) | 6 (14%) | 37 (84%) | 1 (2.3%) | 18 (41%) | 26 (59%) |
| No | 3 (100%) | 3 (100%) | 0 (0%) | 0 (0%) | 1 (33%) | 2 (67%) | 0 (0%) | 3 (100%) | 0 (0%) | 1 (33%) | 2 (67%) |
| Yes | 106 (100%) | 30 (28%) | 54 (51%) | 22 (21%) | 62 (58%) | 44 (42%) | 15 (14%) | 88 (84%) | 2 (1.9%) | 58 (55%) | 48 (45%) |
| Unknown | 1 | 0 | 0 | 1 | 1 | 0 | 0 | 1 | 0 | 1 | 0 |
| **Was this pain acceptable for you in the context of this study?** | | | | | | | | | | | |
| No, not acceptable | 2 (100%) | 1 (50%) | 1 (50%) | 0 (0%) | 2 (100%) | 0 (0%) | 1 (50%) | 1 (50%) | 0 (0%) | 1 (50%) | 1 (50%) |
| Yes, acceptable | 1 (100%) | 1 (100%) | 0 (0%) | 0 (0%) | 1 (100%) | 0 (0%) | 0 (0%) | 0 (0%) | 1 (100%) | 1 (100%) | 0 (0%) |
| Yes, partially acceptable | 150 (100%) | 46 (31%) | 76 (51%) | 28 (19%) | 87 (58%) | 63 (42%) | 20 (13%) | 127 (85%) | 2 (1.3%) | 76 (51%) | 74 (49%) |
| Unknown | 1 | 0 | 1 | 0 | 0 | 1 | 0 | 1 | 0 | 0 | 1 |
| **How much did the blood self-sampling hurt on a scale from 0 (no pain at all) to 10 (worst imaginable pain)?** | | | | | | | | | | | |
|  | 1.00 (1.00, 3.00) | 1.00 (1.00, 3.00) | 2.00 (1.00, 3.00) | 1.00 (1.00, 2.00) | 1.00 (1.00, 3.00) | 1.00 (1.00, 2.00) | 1.00 (1.00, 2.00) | 1.00 (1.00, 2.50) | 4.00 (1.00, 4.00) | 2.00 (1.00, 3.00) | 1.00 (1.00, 2.00) |
| Unknown | 1 | 0 | 1 | 0 | 0 | 1 | 0 | 1 | 0 | 0 | 1 |
| **How many of the enclosed lancets did you use?** | | | | | | | | | | | |
| 1 | 69 (100%) | 19 (28%) | 34 (49%) | 16 (23%) | 41 (59%) | 28 (41%) | 12 (57%) | 56 (44%) | 1 (33%) | 31 (40%) | 38 (50%) |
| 2 | 84 (100%) | 29 (35%) | 43 (51%) | 12 (14%) | 48 (57%) | 36 (43%) | 9 (43%) | 72 (56%) | 2 (67%) | 46 (60%) | 38 (50%) |
| Unknown | 1 | 0 | 1 | 0 | 1 | 0 | 0 | 1 | 0 | 1 | 0 |
| **Which method(s) did you use to improve blood circulation before taking the blood sample?** | | | | | | | | | | | |
| No | 15 (100%) | 4 (27%) | 5 (33%) | 6 (40%) | 6 (40%) | 9 (60%) | 2 (14%) | 12 (86%) | 0 (0%) | 10 (67%) | 5 (33%) |
| At least one method | 138 (100%) | 43 (31%) | 73 (53%) | 22 (16%) | 84 (61%) | 54 (39%) | 19 (14%) | 116 (84%) | 3 (2.2%) | 68 (49%) | 70 (51%) |
| Not reported | 1 (100%) | 1 (100%) | 0 (0%) | 0 (0%) | 0 (0%) | 1 (100%) | 0 (0%) | 1 (100%) | 0 (0%) | 0 (0%) | 1 (100%) |
| Unknown | 0 | 0 | 0 | 0 | 0 | 0 | 0 | 0 | 0 | 0 | 0 |
| Method: hold hand/ finger under very hot water | 25 (100%) | 6 (24%) | 15 (60%) | 4 (16%) | 16 (64%) | 9 (36%) | 3 (12%) | 21 (84%) | 1 (4.0%) | 13 (52%) | 12 (48%) |
| Unknown | 0 | 0 | 0 | 0 | 0 | 0 | 0 | 0 | 0 | 0 | 0 |
| Method: shake hand strongly | 73 (100%) | 25 (34%) | 33 (45%) | 15 (21%) | 51 (70%) | 22 (30%) | 8 (11%) | 62 (85%) | 3 (4.1%) | 39 (53%) | 34 (47%) |
| Unknown | 0 | 0 | 0 | 0 | 0 | 0 | 0 | 0 | 0 | 0 | 0 |
| Method:  Massage finger | 102 (100%) | 35 (34%) | 51 (50%) | 16 (16%) | 62 (61%) | 40 (39%) | 15 (15%) | 86 (84%) | 1 (1.0%) | 48 (47%) | 54 (53%) |
| Unknown | 0 | 0 | 0 | 0 | 0 | 0 | 0 | 0 | 0 | 0 | 0 |
| Method:  “Pumps” (open and close fist) | 76 (100%) | 29 (38%) | 36 (47%) | 11 (14%) | 50 (66%) | 26 (34%) | 6 (7.9%) | 68 (89%) | 2 (2.6%) | 39 (51%) | 37 (49%) |
| Unknown | 0 | 0 | 0 | 0 | 0 | 0 | 0 | 0 | 0 | 0 | 0 |
| Method:  Move arm quickly back and forth while hanging down | 37 (100%) | 8 (22%) | 22 (59%) | 7 (19%) | 21 (57%) | 16 (43%) | 2 (5.4%) | 35 (95%) | 0 (0%) | 23 (62%) | 14 (38%) |
| Unknown | 0 | 0 | 0 | 0 | 0 | 0 | 0 | 0 | 0 | 0 | 0 |
| **Have you wiped away the first drop of blood?** | | | | | | | | | | | |
| No | 6 (100%) | 3 (50%) | 3 (50%) | 0 (0%) | 3 (50%) | 3 (50%) | 1 (17%) | 5 (83%) | 0 (0%) | 3 (50%) | 3 (50%) |
| Yes | 144 (100%) | 43 (30%) | 73 (51%) | 28 (19%) | 86 (60%) | 58 (40%) | 20 (14%) | 121 (84%) | 3 (2.1%) | 73 (51%) | 71 (49%) |
| Don’t know | 4 (100%) | 2 (50%) | 2 (50%) | 0 (0%) | 1 (25%) | 3 (75%) | 0 (0%) | 3 (100%) | 0 (0%) | 2 (50%) | 2 (50%) |
| Unknown | 0 | 0 | 0 | 0 | 0 | 0 | 0 | 0 | 0 | 0 | 0 |
| **How many drops of blood did you need for this?** | | | | | | | | | | | |
| 1 | 4 (100%) | 2 (50%) | 2 (50%) | 0 (0%) | 1 (25%) | 3 (75%) | 0 (0%) | 4 (100%) | 0 (0%) | 2 (50%) | 2 (50%) |
| 2-3 | 48 (100%) | 13 (27%) | 26 (54%) | 9 (19%) | 26 (54%) | 22 (46%) | 11 (23%) | 36 (75%) | 1 (2.1%) | 19 (40%) | 29 (60%) |
| 3+ | 67 (100%) | 17 (25%) | 37 (55%) | 13 (19%) | 48 (72%) | 19 (28%) | 8 (12%) | 57 (86%) | 1 (1.5%) | 38 (57%) | 29 (43%) |
| Don't know | 26 (100%) | 12 (46%) | 10 (38%) | 4 (15%) | 10 (38%) | 16 (62%) | 1 (3.8%) | 24 (92%) | 1 (3.8%) | 14 (54%) | 12 (46%) |
| Unknown | 9 | 4 | 3 | 2 | 5 | 4 | 1 | 8 | 0 | 5 | 4 |
| **To what extent was the manual understandable for you?** | | | | | | | | | | | |
| Easy understandable | 142 (100%) | 44 (31%) | 72 (51%) | 26 (18%) | 84 (59%) | 58 (41%) | 19 (13%) | 119 (84%) | 3 (2.1%) | 70 (49%) | 72 (51%) |
| Partially understandable | 12 (100%) | 4 (33%) | 6 (50%) | 2 (17%) | 6 (50%) | 6 (50%) | 2 (17%) | 10 (83%) | 0 (0%) | 8 (67%) | 4 (33%) |
| Unknown | 0 | 0 | 0 | 0 | 0 | 0 | 0 | 0 | 0 | 0 | 0 |
| **Has the puncture site bled longer and/or heavily?** | | | | | | | | | | | |
| No | 132  (100%) | 40  (30%) | 66  (50%) | 26  (20%) | 75  (78%) | 57  (22%) | 17  (13%) | 112  (85%) | 3  (3.0%) | 68  (52%) | 64  (49%) |
| Yes | 18 (100%) | 7 (39%) | 9 (50%) | 2 (11%) | 14 (78%) | 4 (22%) | 3 (18%) | 14 (82%) | 0 (0%) | 7 (39%) | 11 (61%) |
| Unknown | 4 | 1 | 3 | 0 | 1 | 3 | 1 | 3 | 0 | 3 | 1 |
| *^1^*n (%); Median (Q1, Q3);  ^2^Question: Have you ever –prior to this study- taken blood sample? | | | | | | | | |  |  |  |

S2 Appendix Table 2. Non-responder characteristics

| **Characteristic** | **Overall** | **Stratification** | | | | | | |
| --- | --- | --- | --- | --- | --- | --- | --- | --- |
|  |  | **Age** | | | **Gender/ sex** | | **Education** | |
|  | N = 35*^1^* | **<30** N = 9*^1^* | **30-49** N = 16*^1^* | **≥50** N = 10*^1^* | **Female** N = 23*^1^* | **Male** N = 11*^1^* | **Lower than ISCED level** N = 8*^1^* | **ISCED level 3** N = 26*^1^* |
| **I have physical disabilities/ health restrictions that do not allow me to participate.** | | | | | | | | |
| It applies to … | 9 (100%) | 1 (11%) | 4 (44%) | 4 (44%) | 5 (56%) | 4 (44%) | 3 (33%) | 6 (67%) |
| Unknown | 0 | 0 | 0 | 0 | 0 | 0 | 0 | 0 |
| **Participating is too time-consuming.** | | | | | | | | |
| It applies to … | 1 (100%) | 1 (100%) | 0 (0%) | 0 (0%) | 0 (0%) | 1 (100%) | 0 (0%) | 1 (100%) |
| Unknown | 0 | 0 | 0 | 0 | 0 | 0 | 0 | 0 |
| **I generally have inhibitions regarding drawing of blood samples.** | | | | | | | | |
| It applies to … | 13 (100%) | 6 (46%) | 6 (46%) | 1 (7.7%) | 8 (67%) | 4 (33%) | 4 (31%) | 9 (69%) |
| Unknown | 0 | 0 | 0 | 0 | 0 | 0 | 0 | 0 |
| **I am afraid of blood self-sampling.** | | | | | | | | |
| It applies to … | 13 (100%) | 3 (23%) | 8 (62%) | 2 (15%) | 12 (100%) | 0 (0%) | 5 (38%) | 8 (62%) |
| Unknown | 0 | 0 | 0 | 0 | 0 | 0 | 0 | 0 |
| **I don't see the needs and benefits of the study.** | | | | | | | | |
| It applies to … | 1 (100%) | 0 (0%) | 0 (0%) | 1 (100%) | 0 (0%) | 1 (100%) | 0 (0%) | 1 (100%) |
| Unknown | 0 | 0 | 0 | 0 | 0 | 0 | 0 | 0 |
| **I don't find the instrument for blood self-sampling very trustworthy.** | | | | | | | | |
| Does not apply | 35 (100%) | 9 (26%) | 16 (46%) | 10 (29%) | 23 (68%) | 11 (32%) | 8 (24%) | 26 (76%) |
| Unknown | 0 | 0 | 0 | 0 | 0 | 0 | 0 | 0 |
| **I have concerns about data protection** | | | | | | | | |
| Does not apply | 35 (100%) | 9 (26%) | 16 (46%) | 10 (29%) | 23 (68%) | 11 (32%) | 8 (24%) | 26 (76%) |
| Unknown | 0 | 0 | 0 | 0 | 0 | 0 | 0 | 0 |
| **I generally don't participate in surveys or studies** | | | | | | | | |
| It applies to … | 1 (100%) | 0 (0%) | 1 (100%) | 0 (0%) | 0 (0%) | 1 (100%) | 8 (24%) | 26 (76%) |
| Unknown | 0 | 0 | 0 | 0 | 0 | 0 | 0 | 0 |
| **I generally don't like to participate in studies, that are carried out at my workplace.** | | | | | | | | |
| It applies to … | 2 (100%) | 0 (0%) | 1 (50%) | 1 (50%) | 1 (50%) | 1 (50%) | 0 (0%) | 1 (100%) |
| Unknown | 0 | 0 | 0 | 0 | 0 | 0 | 0 | 0 |
| **Another reason.** | | | | | | | | |
| It applies to … | 8 (100%) | 1 (13%) | 4 (50%) | 3 (38%) | 7 (88%) | 1 (13%) | 1 (13%) | 7 (88%) |
| Unknown | 0 | 0 | 0 | 0 | 0 | 0 | 0 | 0 |
| *^1^*n (%) | | | | | | | | |

S3 Appendix. Study specific instructions. Manual containing study specific instructions. (PDF)

S4 Appendix. Questionnaire. Questionnaire used to assess feasibility and acceptability of self-sampling with the HemaSpot device. (PDF)

S5 Appendix. Non-responder questionnaire. Questionnaire used to assess reasons for non-participation. (PDF)
